# Genomic and probiotic characterization of SJP-SNU strain of *Pichia kudriavzevii*

**DOI:** 10.1186/s13568-018-0609-0

**Published:** 2018-05-17

**Authors:** Seung-Min Hong, Hyuk-Joon Kwon, Se-Joon Park, Won-Jin Seong, Ilhwan Kim, Jae-Hong Kim

**Affiliations:** 1 Laboratory of Avian Diseases, College of Veterinary Medicine and BK21 PLUS for Veterinary Science, Seoul National University, 1, Gwanak-ro, Gwanak-gu, Seoul, 08826 Republic of Korea; 2 Laboratory of Poultry Production Medicine, College of Veterinary Medicine and BK21 PLUS for Veterinary Science, Research Institute for Veterinary Science, Seoul National University, Seoul, 08826 Republic of Korea; 3 Healingbio Co., Seoul, 04393 Republic of Korea; 4 Center for Infectious Diseases, Korean National Institute of Health, Osong, 28159 Republic of Korea

**Keywords:** Novel yeast, Probiotics, De novo sequencing, Comparative genomics, Pathogenicity, Evolution

## Abstract

The yeast strain SJP-SNU was investigated as a probiotic and was characterized with respect to growth temperature, bile salt resistance, hydrogen sulfide reducing activity, intestinal survival ability and chicken embryo pathogenicity. In addition, we determined the complete genomic and mitochondrial sequences of SJP-SNU and conducted comparative genomics analyses. SJP-SNU grew rapidly at 37 °C and formed colonies on MacConkey agar containing bile salt. SJP-SNU reduced hydrogen sulfide produced by *Salmonella* serotype Enteritidis and, after being fed to 4-week-old chickens, could be isolated from cecal feces. SJP-SNU did not cause mortality in 10-day-old chicken embryos. From 13 initial contigs, 11 were finally assembled and represented 10 chromosomal sequences and 1 mitochondrial DNA sequence. Comparative genomic analyses revealed that SJP-SNU was a strain of *Pichia kudriavzevii*. Although SJP-SNU possesses pathogenicity-related genes, they showed very low amino acid sequence identities to those of *Candida albicans*. Furthermore, SJP-SNU possessed useful genes, such as phytases and cellulase. Thus, SJP-SNU is a useful yeast possessing the basic traits of a probiotic, and further studies to demonstrate its efficacy as a probiotic in the future may be warranted.

**Electronic supplementary material:**

The online version of this article (10.1186/s13568-018-0609-0) contains supplementary material, which is available to authorized users.

## Introduction

Many yeasts are present in fermented materials, feces and various environmental sources, a number of which have been used in the production of fermented foods, wine and biofuels. *Pichia pastoris* has been attempted to be used for single cell protein as animal feed additive, while *Saccharomyces cerevisiae* has been used as a probiotic for farm animals (Ahmad et al. 2014; Chaucheyras-Durand and Fonty 2001). *Saccharomyces boulardii* has been used as a probiotic in humans and farm animals due to its antibacterial and anti-diarrheal activities (Baum et al. 2002; Kelesidis and Pothoulakis 2012). Compared with *S. cerevisiae*, *S. boulardii* grows at body temperature and may be the preferred choice for use as a probiotic in animals. Probiotics were defined as live microorganisms which confer a health benefit on the host and include bacteria, *Lactobacillus*, *Bifidobacterium* etc. and yeasts (Ref. FAO/WHO. Health and nutritional properties of probiotics in food including powder milk with live lactic acid bacteria. Córdoba: FAO/WHO; 2001).

Many fungi have been classified on the basis of morphology, but molecular taxonomy has been used in the classification of yeasts. Recently, comparative genomic studies have unraveled the phylogenetic relationships and evolutionary mechanisms of yeasts. Yeasts have enriched their genetic material by genome duplication, hybridization and the acquisition of foreign genes and diversified their genomes via extensive gene loss and loss of heterozygosity (Butler et al. 2009; Dujon 2010; Gojkovic et al. 2004; Greig et al. 2002; Marinoni et al. 1999; Wolfe and Shields 1997). Yeasts can replicate by sexual and asexual reproduction but have relatively low outcrossing rates (Tsai et al. 2008).

In the present study, we isolated a yeast SJP-SNU from the decomposed leaves of plants and characterized its basic traits for potential use as a probiotic. We also determined the yeast’s complete genome sequence and conducted comparative genomic analyses to understand its phylogenetic and taxonomic relationships with other yeasts and to identify genes related to pathogenicity, metabolism and other useful phenotypic-related genes.

## Materials and methods

### Yeast, bacteria and media

The SJP-SNU (KCTC 12756BP) strain was isolated from fermented plants and developed for use as a probiotic by Healingbio Co. (14, Jangmun-ro, Yongsan-gu, Seoul, 04393, Korea). The SJP-SNU strain was cultured using YM agar and broth (BD, Sparks, MD, USA) at 37 °C. *Saccharomyces cerevisiae* (KCTC 7904) and *Saccharomyces cerevisiae var. boulardii* CNCM I-1079 (*S. boulardii*, Lallemand Inc., Quebec, Canada) were also cultured using YM agar and broth (BD). *Salmonella* serotype Enteritidis (SE50), isolated from a chicken, was cultured using MacConkey and TSI agars, and TSA broth (BD). The SJP-SNU strain was deposited to the Korean Collection for Type Cultures (KCTC-12756BP) in Korea.

### Comparison of bile salt resistance and anaerobic growth at 25 and 37 °C

For successful settle-down of probiotics in gastrointestinal tract resistance to bile salt is one of necessary requirements (Klaenhammer and Kullen 1999). *S. boulardii* is known to grow at 37 °C and has been used as a probiotic in humans. To compare the bile salt resistance and growth efficiency at 37 °C of SJP-SNU and *S. boulardii*, the strains were cultured on MacConkey agar plates containing bile salt at 37 °C, with colony formation being observed for 5 days. To assess the anaerobic growth of SJP-SNU, the strain was streaked onto YM agar plates and incubated at 25 or 37 °C in anaerobic jars with GasPaks for 48 h (BD).

### Hydrogen sulfide (H_2_S) reduction test

H_2_S is a toxic gas generated by bacteria in the intestine. To test the H_2_S reduction ability of SJP-SNU, first *Salmonella* Enteritidis (SE, 4.3 × 10^9^ cfu/ml) was 10-fold serially diluted, and each dilution was inoculated into TSI (Triple sugar iron) agar by stabbing. The minimum bacterial count that generated H_2_S was determined to be 10^6^ cfu/ml. Next, undiluted cultures of SJP-SNU (8.8 × 10^9^ cfu/ml) and *S. boulardii* (2.0 × 10^8^ cfu/ml) were 10-fold diluted in PBS. Finally, 450 µl of each yeast dilution and 50 µl of H_2_S-producing *Salmonella* serotype Enteritidis (10^6^ cfu/ml) were mixed and inoculated into TSI agar and immediately incubated overnight at 37 °C.

### Intestinal viability test

*Saccharomyces cerevisiae* has been employed as a probiotic for farm animals. To compare the intestinal viability of *S. cerevisiae* and SJP-SNU, fifteen 4-week-old specific pathogen free (SPF) chickens (Valo, USA) were grouped into three groups (two inoculation and control groups), and orally inoculated with 10^7^ cfu/ml/chicken/day of yeast for 5 days. At 7 days post-inoculation (dpi), cecal feces were collected and diluted by 10-fold with sterilized PBS. Diluted feces were spread onto YM agar plates containing antibiotics (ampicillin 50 µg/ml, tetracycline 50 µg/ml, gentamicin 50 µg/ml, kanamycin 50 µg/ml, and streptomycin 50 µg/ml) and were incubated at 37 °C or 25 °C for growth of SJP-SNU and *S. cerevisiae*, respectively. The cfu/g of feces was calculated by multiplying the dilution factor and the number of cfu. Animal protocols used in the study were approved by the Biopoa Co., Ltd. institutional IACUC and performed in accordance with all relevant policies.

### Chicken embryo pathogenicity test

To evaluate the pathogenicity of yeast or fungi chicken embryos were inoculated onto chorioallantoic membrane (CAM) but we selected more aggressive route into allantoic cavity (Alexander 1989; Jacobsen et al. 2010). Six 10-day-old SPF embryonated chicken eggs (ECEs, Charles River Laboratories, North Franklin, USA) were inoculated with 10^5^ cfu/egg of SJP-SNU or PBS via the allantoic cavity and were candled twice a day for 60 h during incubation at 37 °C to assess embryo survival. After 3 days of incubation, ECEs were chilled at 4 °C overnight and embryos lesions were observed.

### Genome sequencing and assembly

Total DNA of an overnight culture of SJP-SNU was extracted using a DNeasy^®^ Blood & tissue kit (QIAGEN), and PacBio RS II single molecule real time (SMRT) sequencing of SJP-SNU was performed (Theragen ETS, Seongnam, Korea). Briefly, 10 μg of yeast genomic DNA was sheared with a Covaris^®^ g-TUBE^®^ device and size-selection for 15–50 kb was performed with a BluePippin system (0.75% DF Marker S1 high-pass 15–20 kb), both done according to the manufacturer’s protocols. SMRTbell template libraries were subsequently prepared using the commercial Template Preparation Kit from Pacific Biosciences Inc. and involved the sequential steps of DNA end repair, adapter ligation and exonuclease digestion of incompletely ligated products. Next, 0.83 nM of the libraries were later annealed to the sequencing primers followed by binding to 50 nM of P4 DNA polymerase, provided in the Template Binding Kit from Pacific Biosciences Inc. For enhanced loading efficiency, 15 pM of the bound complexes were immobilized onto Magbeads (Pacific Biosciences Inc.) prior to loading into the sequencing zero-mode waveguides (ZMWs). The duration for the sequence collection was set at 180 min using the stage start option. Reads with a length of less than 50 bp were filtered out upon acquisition of the sequencing data, and the minimum polymerase read quality was set at 0.75. The SMRT sequencing data were assembled de novo using the FALCON and HGAP3 software pipelines, and the results were merged and reconciled with GARM metaassembler.

### Generation and sequencing of a HiSeq DNAPCR-free library

Each sequenced sample was prepared according to the Illumina protocols. The quantification of DNA and the DNA quality was measured by PicoGreen and Nanodrop. Briefly, one microgram of genomic DNA was fragmented by a Covaris device to obtain 350 bp-sized fragments. The fragmented DNA was blunt-ended and phosphorylated, followed by end repair, and the appropriate library size was selected using different ratios of the sample purification beads. A single ‘A’ was ligated to the 3′ ends of DNA fragments, then Illumina adapters were ligated to the fragments. The final ligated product was then quantified using qPCR according to the qPCR Quantification Protocol Guide and was validated using an Agilent Technologies 2100 Bioanalyzer. (Agilent Technologies, Palo Alto CA, USA). Sequencing was performed using a HiSeq™ 2000 platform (Illumina, San Diego, USA).

The genome sequence data obtained from SMRT sequencing were used for all genome analyses performed in the present study. However, only data obtained from the Illumina HiSeq II was used for pathogenic gene analyses due to its high sequence fidelity.

### Prediction of repeats and non-coding RNAs (ncRNAs)

For the repeat composition analysis, we used reference based (RepeatMasker ver. 4.0.7 and RepBase (14,031) library; Institute for System Biology) and de novo based (RepeatModeler, ver. 1.0.8; Institute for System Biology) methods, and the results were combined. Simple sequence repeats (SSRs) were searched in the sequenced genome of SJP-SNU by using SSR Finder (minimal number of repeats was 5). The tRNA sequences were predicted by comparing nucleotide homology with tRNAscan-SE2.0 (http://lowelab.ucsc.edu/tRNAscan-SE/). Small nuclear RNA (snRNA) structure and sequence similarities were assessed using the INFERNAL Tool and rfam database (http://eddylab.org/infernal/). Ribosomal RNA (rRNA) sequences were searched by comparing homology with BLAST.

### Prediction and annotation of gene structure

The proteins in the genomes of related yeasts identified by taxonomy profiling were extracted from the NCBI non-redundant (nr) protein database and genes were predicted using the exonerate tool. The final gene set was established by combining cording partial information of combining exonerate tool and intron information of protein hint with gene model of AUGUSTUS (Stanke et al. 2004). Gene annotation was performed by homology search of gene set against UniProt, NCBI nr and InterProScan databases.

### In silico characterization of pathogenic and biologically important genes

The pathogenicity-related genes of *Candida albicans* were selected and nucleotide sequences were collected from GenBank databases (Navarro-Garcia et al. 2001). Nucleotide sequences of collected genes were translated with the program BioEdit. By searching for the pathogenic and biologically important genes and protein names in the gene annotation data of SJP-SNU, homologous genes were collected to compare amino acid sequences using the BLASTP search program.

## Results

### Comparison of growth temperature, anaerobic growth and bile salt resistance

**Fig. 1:**
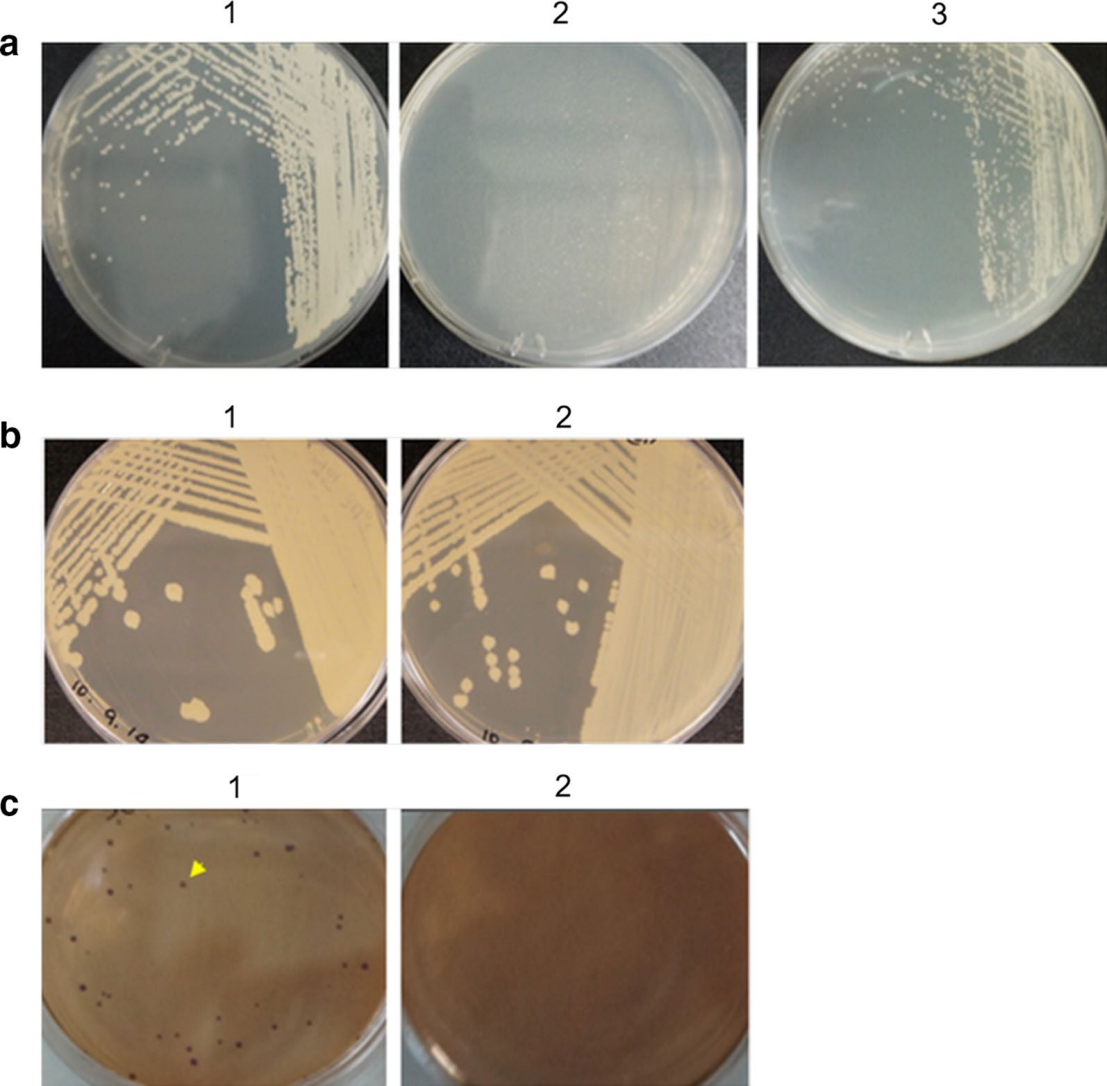
Growth characteristics of SJP-SNU. **a** Comparison of growth efficiency of SJP-SNU and *S. boulardii* at 37 °C on YM agar plates (1: SJP-SNU after 18 h, 2: *S. boulardii* after 18 h., 3: *S. boulardii* after 32 h.). **b** Anaerobic growth of SJP-SNU on YM agar plates (1: Aerobic condition at 37 °C for 48 h., 2: Anaerobic condition at 37 °C for 48 h.). **c** Comparison of bile salt resistance of SJP-SNU and *S. boulardii* cultured at 37 °C on MacConkey agar plates for 5 days (1: SJP-SNU, 2: *S. boulardii*)

The growth of SJP-SNU at 37 °C was compared with *S. boulardii*. SJP-SNU formed visible large colonies after 18 h of incubation while *S. boulardii* formed small colonies only after 32 h incubation. Therefore, the growth rate of SJP-SNU was greater than *S. boulardii* at 37 °C (Fig. 1a).

Because *S. cerevisiae* is known to grow under anaerobic conditions, the anaerobic growth of SJP-SNU was compared with *S. cerevisiae*. SJP-SNU and *S. cerevisiae* were incubated anaerobically at 37 and 25 °C, respectively, for 48 h on YM plates. SJP-SNU formed large visible colonies under both aerobic and anaerobic conditions (Fig. 1b).

SJP-SNU and *S. boulardii* were cultured on MacConkey agar plates at 37 °C for 5 days. While SJP-SNU formed visible colonies, *S. boulardii* did not. Thus, the growth of SJP-SNU was retarded but not completely inhibited (Fig. 1c).

### Hydrogen sulfide (H_2_S) reduction ability of SJP-SNU strain

**Fig. 2:**
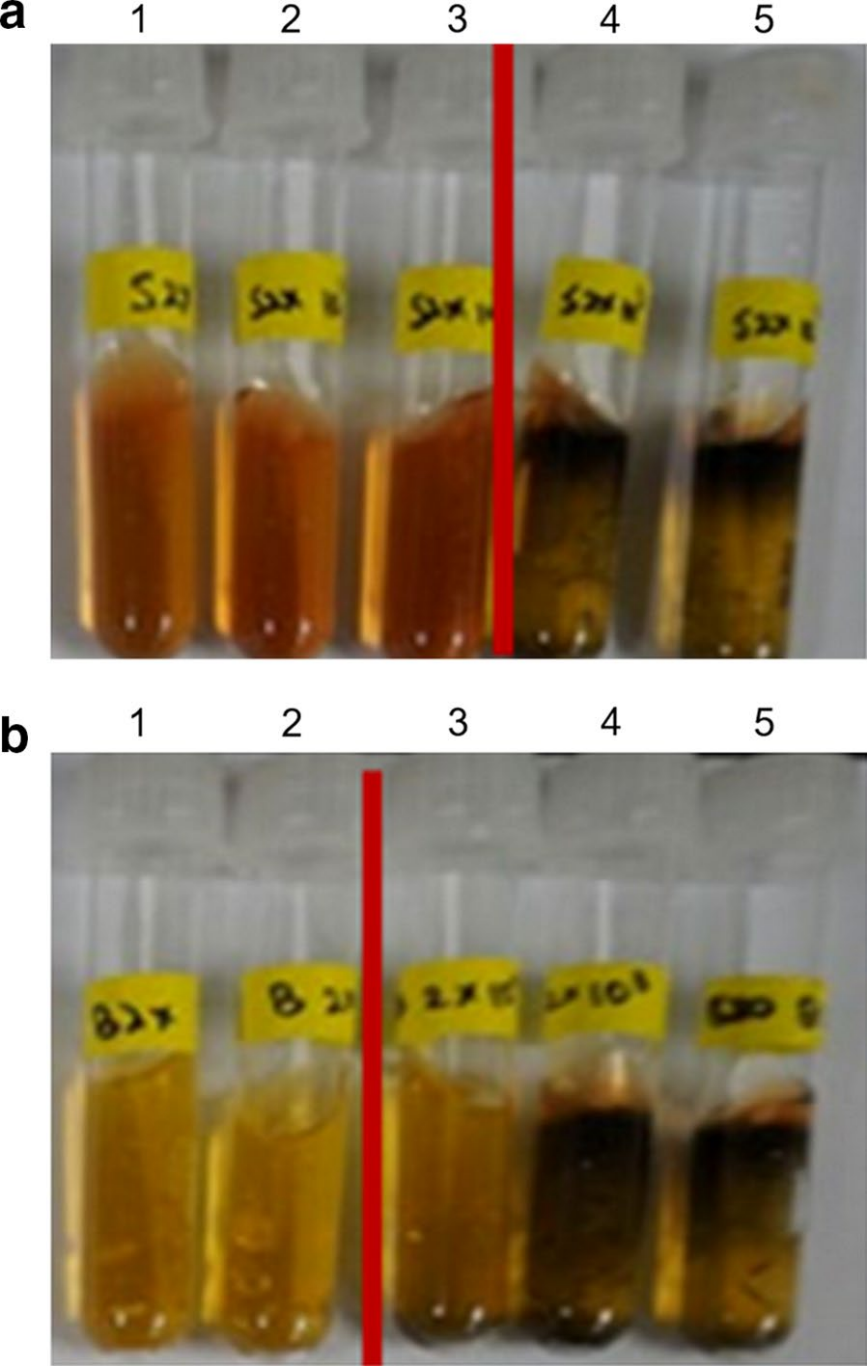
H_2_S removal activity of SJP-SNU and *S. boulardii*. SJP-SNU (8.8 × 10^9^ cfu/ml, **a**) and *S. boulardii* (2 × 10^8^ cfu/ml, **b**) were diluted 10-fold (1: 1, 2: 10^−1^, 3: 10^−2^, 4: 10^−3^, 5: 10^−4^) and co-cultured with H_2_S-prodcing *Salmonella* serotype Enteritidis (10^6^ cfu/ml) in TSI agar medium at 37 °C for 18 h. The red bar separates H_2_S-negative (left) and H_2_S-positive (right) tubes

TSI medium contains ferrous sulfate as an indicator of H_2_S production by inoculated bacteria. SJP-SNU and *S. boulardii* were tested for H_2_S-reducing activity. Co-cultures of either SJP-SNU or *S. boulardii* with H_2_S-producing *Salmonella* Enteritidis decreased the blackish discoloration of the TSI media. The 100-fold- (8.8 × 10^7^) and 10-fold-diluted (2.0 × 10^7^) SJP and *S. boulardii*, respectively, suppressed completely the discoloration of media (Fig. 2).

### Comparison of intestinal viability of *S. cerevisiae* and SJP-SNU in chicken

Probiotic microorganisms need to survive in the intestines to provide positive effects for hosts. We orally administered 10^7^ cfu/ml of SJP-SNU and *S. cerevisiae* to 4-week-old SPF chickens daily for 5 days and the number of yeast in feces were counted using YM agar plates containing multiple antibiotics. No yeasts were isolated in the cecal feces of the negative control and *S. cerevisiae*-administered chickens, whereas yeasts were isolated from the cecal feces of SJP-SNU-administered chickens (Table 1).

**Table 1:** Intestinal viability of *S. cerevisiae* and SJP-SNU in chickens

Group	Colony forming unit/gram of feces					
	1	2	3	4	5	Median
*S. cerevisiae*	0	0	0	0	0	0
SJP-SNU	2.8 × 10^4^	7.5 × 10^5^	1.1 × 10^5^	1.9 × 10^5^	1.3 × 10^5^	1.3 × 10^5^
Control	0	0	0	0	–	0

### Pathogenicity of SJP-SNU in chicken embryos

The pathogenicity of SJP-SNU was tested in embryonated chicken eggs (ECEs). The SJP-SNU strain was inoculated via the allantoic cavity route and did not cause mortality of embryos for 3 days and the embryos did not show any congestion, hemorrhaging or body atrophy. Thus, SJP-SNU had no pathogenic effect on chicken embryos.

### Structure and functional annotation of the SJP-SNU genome

The PacBio sequencing and assembly pipelines generated 13 contigs of 11,005,966 bases (depth of 78.6 and a N50 of 1,367,155 bp) and we conducted sequence comparisons by querying paired contigs in BLATN programs. We observed that contig 13 (22,460 bp) was a part of contig 12 (56,499 bp) and more than the first half (36,004 bp) of contig 12 overlapped the 3′ end of contig 1. We joined the remaining half of contig 12 to contig 1. In addition, we found overlapping sequences at the both ends of contig 11 and removed the repeated sequences at the 3′-end (24,003 bp). The final size (10,923,756 bp) and GC content (38.2%) of the 11 contigs were summarized in Table 2. The genome sequence of SJP-SNU strain was deposited in Genebank under the accession number (SUB2596880; PRJNA383123; SAMN06675446). In total, 3878 gene models (3713 of unique genes and 165 isoforms) were predicted and 3590 genes were annotated. The average gene length was 1320 bp and the total bases of gene models was 5.12 Mbp (46.53% of the draft genome). The number of exons was 6012 (1.55 exon/gene on average) and the average exon length was 716 bp. The number of introns was 2134 (0.55 intron/gene on average) and the average exon length was 380 bp. The exons and introns covered 39.15 and 7.38% of the draft genome, respectively.

**Table 2:** Lengths and GC contents of contigs

Contig	Length (bp)	GC_Count	GC_content
1	1,536,420	581,626	0.383677
2	1,500,387	568,366	0.378813
3	1,371,280	527,301	0.384532
4	1,367,155	530,165	0.387787
5	1,237,440	479,634	0.387602
6	1,194,786	456,559	0.382126
7	1,179,339	447,829	0.379729
8	1,160,046	446,046	0.384507
9	198,455	76,490	0.385427
10	127,320	51,058	0.401021
11	74,874	12,228	0.163314
Total	10,947,502	4,177,302	0.381575

Retrotransposons and unclassified and simple repeats were predicted and covered 2.87% of the genome (Additional file 1: Table S1). The copy numbers of ribosomal RNA (rRNA), transfer RNA (tRNA), and small nuclear RNA (snRNA) were 29, 209 and 257, and covered 0.32, 0.14 and 0.19% of genome, respectively (Additional file 1: Table S2). The genes related to the biological characteristics and pathogenicity of SJP-SNU were deposited in Genebank under the accession number (Tables 3 and 4).

**Table 3:** Important genes for prediction of biological traits of SJP-SNU

Activity	Symbol of gene	Gene of SJP-SNU	Accession nos.
Anaerobiosis	*ARG82*	i04794	MF580678
	*ARV1*	i01749	MF580679
	*CAX4*	i01146	MF580680
	*CDC40*	i02538	MF580681
	*DBP7*	i04141	MF580682
	*DRS2*	i04217	MF580683
	*FZO1*	i01570	MF580684
	*GMP1*	i00484	MF580685
	*HFI1*	i01065	MF580686
	*MRPL10*	i04329	MF580687
	*NPT1*	i02351	MF580688
	*SIN4*	i00449	MF580689
	*SNF1*	i04534	MF580690
	*SPT20*	i04515	MF580691
	*SPT80*	i04515	MF580691
Cellulase	*EXG_PICAN*	i03243	MF580692
		i04958	MF580693
	*YBR056* *W*	i02448	MF580694
	*YIR007* *W*	i03386	MF580695
Bile acid permease	*YBT1*	i03062	MF740757
Phytase	*PHYA*	i01331	MF580696
		i01362	MF580697
		i01364	MF580698
	*PHYB*	i01367	MF580699
*Endo*-1,3(4)-beta-glucanase	*ACF2*	i02149	MF580700
Glucoamylase	*GLU1*	i03516	MF580701
Mating	*STE2*	i02542	MF740758
	*STE3*	i01062	MF740759

**Table 4:** Pathogenicity-related genes of SJP-SNU

Activity	Gene of *C. albicans*	Gene of SJP-SNU	BLASTP information^a^	Accession numbers
Fatty acid synthase	*FAS2*	i01491	100/71/0.0	MF580702
Type I membrane ferroxidase	*FET3*	i01900	98/54/0.0	MF580703
		i02637	95/46/0.0	MF580704
High-affinity iron permease	*FTR1*	i00027	93/67/5e−174^a^	MF580705
Haemosynthesis	*HEM3*	i01397	97/59/2e−138	MF580706
Amino acid biosynthesis	*HIS1*	i04163	100/67/8e−150	MF580707
	*LEU2*	i03643	98/77/0.0	MF580708
Orotidine-5′-phosphate decarboxylase	*URA3*	i00907	95/74/2e−154	MF580709
Trehalose-phosphate synthase	*TPS1*	01960	95/70/0.0	MF580722
Chitin synthase	*CHS2*	i03074	79/42/0.0	MF740748
	*CHS3*	i01942	93/56/0.0	MF580726
Glucosamine-6-phosphate acetyltransferase	*GNA1*	i04566	96/44/5e−40	MF580710
β-Glucosyltransferase	*BGL2*	i01739	92/61/5e−134	MF580713
GTPase	*RSR1*	01279/i04119	99/59/1e−104	MF580718
Transcription factor	*TUP1*	i01055	92/69/0.0	MF580714
MAP kinase	*CEK1*	i00979	99/76/0.0	MF580715
	*HOG1*	i02281	93/80/0.0	MF580716
	*MKC1*	02283	75/62/7e−170	MF740749
Protein kinase	*CLA4*	i01477	71/52/2e−149	MF740750
	*VPS34*	i00437	88/43/6e−133	MF740751
α-Mannosyltransferase	*MNT1*	i02982	98/56/1e−179	MF580711
	*PMT1*	i04222	85/52/0.0	MF740752
	*PMT6*	i04480	88/41/0.0	MF740753
Endo-1,3-b-glucosidase	*PHR1*	i03503	98/54/0.0	MF580712
	*PHR2*	i03513	84/64/0.0	MF740754
Histidine kinase	*NIK1*	i33342	95/65/0.0	MF580717
	*SLN1*	i02577	80/41/5e−147	MF740755
Catalase	*CTA1*	00609	98/66/0.0	MF580721
Lysophospholipase	*PLB1*	03308/i03297	95/45/7e−171	MF580719
Aspartyl protease	*SAP1*	01553	83/28/7e−29	MF740756
	*SAP2*	02852	95/22/4e−13	MF580723
Topoisomerase	*TOP1*	i02898	94/62/0.0	MF580720

Other pathogenicity-related genes compared in the present study: NA binding protein (MIG1), trehalose-6-phosphate synthase (TPS1), Ferric reductase (RBT2) 5′-AMP-activated protein kinase (SNF1), Extracellular membrane protein (CSP37), Protein kinase (CST20, HST7), Histidine kinase (SSK1), Transcription factor (UME6, TEC1), Glucanase (XOG1), Tyr phosphatase (CPP1), Adherent gene (AAF1), GPI anchored cell wall protein (HWP1, RBT1, 3, WAP1, RBT4), Hyphal growth (HWP1, ECE1), Aspartyl protease (PEP1, SAP3-6), Efflux protein (MDR1)

^a^Coverage/identity (% of amino acid identity to reference sequence of *Candida albicans*)/e value

### Molecular taxonomy of SJP-SNU on the basis of genome sequences

The nucleotide sequences of contigs 1–11 were compared with the genome sequences of yeasts in the GenBank databases. All of the contigs 1–11 showed the highest similarity to *P. kudriavzevii* (taxID 4909) with high e-values. Therefore, SJP-SNU is a strain of *P. kudriavzevii* with basic probiotic traits.

### Characterization of biologically useful genes

Anaerobic growth of probiotic yeasts is required for growth and metabolism in intestinal environments. To date, genes required only under anaerobic conditions have been reported in *S. cerevisiae*, and we searched for these genes in the annotated genes of SJP-SNU (Ishtar Snoek and Yde Steensma 2007). We identified homologous genes that are essential for anaerobic growth, but not-essential for aerobic growth, which are summarized in Table 3. Phytase has been used as a feed additive to increase the digestion of inorganic phosphates in feed, and SJP-SNU possessed four phytase homologs in its genome. Cellulase, endo-1,3(4)-beta-glucanase and glucoamylase cleave the glycosidic bonds of cellulose, cereal d-glucans and starch, respectively, to generate glucose and may improve the feed utilization of farm animals (Casey and Walsh 2004). SJP-SNU possessed four cellulase homologs, one *endo*-1,3(4)-beta-glucanase and one glucoamylase homologs (Table 3). *P. kudriavzevii* homologs that are required for bioethanol production by xylose fermentation were also identified in SJP-SNU (Chan et al. 2012).

### Comparison of pathogenicity-related genes of SJP-SNU to *C. albicans*

To date, various pathogenicity-related genes have been characterized in *C. albicans*, and we compared these to the amino acid sequences of SJP-SNU homologs. Pathogenicity-related genes with more than 90% coverage were selected, and the percentiles of coverage and identity and e-values are summarized in Table 4. SJP-SNU possessed pathogenicity-related genes involved in metabolism (FAS2, FET3, FTR1, HEM3, HIS1, LEU2, URA3, TPS1), cell wall synthesis (CHS3, GNA1, MNT1, PHR1, BGL2), signal transduction (TUP1, CEK1, HOG1) and other functions (CTA1, RSR1, PLB1, SAP2, TOP1), although most of the amino acid sequence identities of the SJP-SNU genes were very low (22–80%) (Table 4).

## Discussion

According to the comparative genomics study SJP was identified as *P. kudriavzevii*. *P. kudriavzevii* has been isolated from fermented foods and fruit juices and is known as *Issatchenkia orientalis* and is an anamorph of *C. krusei* (Arias et al. 2002; Carlotti et al. 1996; Chanprasartsuk et al. 2010; Meroth et al. 2003; Mugula et al. 2003; Zott et al. 2010). *P. kudriavzevii* is heterothallic but is known to be fertile with *P. membranaefaciens, P. scutulata, Candida lambica, C. diversa, C. ingens, C. silvae, C. valida, C. vini, C. norvegensis,* or *Torulopsis inconspicua* (Kurtzman and Smiley 1976).

Live *S. cerevisiae* has been fed to farm animals as a source of vitamins and amino acids and as a probiotic for a long time. *S. cerevisiae* can replicate in anaerobic conditions, although its optimal temperature for active replication and metabolism is lower than the body temperature of farm animals (37–41 °C). The genome of *S. boulardii* is more than 99% similar to the genome to *S. cerevisiae* but it can grow at 37 °C and has been used as a probiotic in humans and farm animals (Kelesidis and Pothoulakis 2012). In the present study, we compared the growth characteristics of SJP-SNU to *S. cerevisiae* and *S. boulardii*. SJP-SNU grew more rapidly than *S. boulardii* on YM and MacConkey agar plates at 37 °C, and both SJP-SNU and *S. cerevisiae* grew in anaerobic conditions. Thus, SJP-SNU possessed the basic essential traits for probiotics usage. These basic essential traits were demonstrated by the re-isolation of a high number of SJP-SNU isolates from cecal feces. In comparison, *S. cerevisiae* was not isolated from cecal feces. To date, various *S. cerevisiae* genes related to anaerobiosis have been reported. Cytoplasmatic dihydroorotate dehydrogenase (DHODase, URA1), involved in de novo pyrimidine biosynthesis, is related to the oxygen-independent growth of *S. cerevisiae* (Gojkovic et al. 2004). Furthermore, dozens of genes essential for anaerobic growth that are not essential for aerobic growth have been reported (Ishtar Snoek and Yde Steensma 2007). Rapid anaerobic growth of yeasts is not common, but *S. cerevisiae* can grow rapidly in anaerobic conditions. SJP-SNU possessed no URA1 but possessed homologs of essential genes for anaerobiosis (Table 3). Therefore, these traits may be related to the presence of SJP-SNU at high numbers in cecal feces.

Ethanol has been used as an antimicrobial agent in foods and is known to inhibit *Listeria monocytogenes* (Oh and Marshall 1993). Yeasts generate ethanol from glucose by alcoholic fermentation under anaerobic conditions. SJP-SNU possess genes involved in alcoholic fermentation using glucose and additional genes (xylose reductase and xylulose kinase genes) for ethanol production with xylose. Therefore, SJP-SNU may produce ethanol in the anaerobic intestines, which may affect microbiota of intestines.

Hydrogen sulfide (H_2_S) is an irritant gas generated under anaerobic conditions by microorganisms in intestines and feces and is a by-product of alcoholic fermentation of yeast. H_2_S is cytotoxic that depletes glutathione, an antioxidant, and increases intracellular iron and reactive oxygen species (Truong et al. 2006). The amount of H_2_S produced by *S. cerevisiae* depends on the strain and nutrient availability, and MET17 plays a role in the conversion of sulfide to cysteine (Cherest and Surdin-Kerjan 1992; Wainwright 1971). SJP-SNU and *S. boulardii* possessed MET17 in their genomes, and their H_2_S reducing activities may be related to MET17. Therefore, the H_2_S reducing activity of SJP-SNU may be valuable trait as a probiotic.

The addition of exogenous cellulase, beta-glucanase and glucoamylase improved digestibility and utilization of feed for farm animals (Kuhad et al. 2011; Mathlouthi et al. 2003; Rojo et al. 2005). SJP-SNU possesses cellulase, beta-glucanase and glucoamylase, and various glucans in ingested feed may be digested efficiently. Phytases catalyze the hydrolysis of phytic acid in feed grain and improve the utilization of digested inorganic phosphorus by farm animals. Therefore, studies on the bioactivities of SJP-SNU phytases and their effect on farm animal productivity may be valuable in the future.

*Candida albicans* is pathogenic, and other opportunistic pathogenic yeasts have been reported. To date, several categories of pathogenicity-related genes of *C. albicans* have been reported (Navarro-Garcia et al. 2001). We collected the homologs of the *C. albicans* pathogenicity-related genes from gene annotation data of SJP-SNU and compared their amino acid sequences. The genes with more than 90% coverage and e-values are summarized in Table 4. Although the e-values are sufficiently high to support that they are homologs of *C. albicans* genes, the amino acid identities are not sufficiently high to extrapolate that they have the same pathogenic roles. SJP-SNU possessed the most similar pathogenicity-related genes to those of *P. kudriavzevii*, but the frequency of *P. kudriavzevii* in clinical cases is less than *C. albicans*. A major concern of *P. kudriavzevii* (*C. krusei*) infections is multi-drug resistance, and the L656C mutation of a glucan synthase (FSK1) is related to resistance to echinocandin (Kahn et al. 2007). SJP-SNU has same FSK1 gene as *P. kudriavzevii*, but does not have the L656C mutation. Although we can not make any conclusions on the opportunistic pathogenicity of SJP-SNU, the lack of mortality and pathogenic lesions of 10-day-old chicken embryos may reflect a low pathogenicity of SJP-SNU. In addition, how the chimeric chromosome composition affects the pathogenicity of SJP-SNU may be the subject future study.

Thus, SJP-SNU is a novel yeast that possesses the basic traits of a probiotic, and the genome data obtained in this study may be useful for understanding the evolution and genotype–phenotype correlation of yeasts.

## Additional files

**Additional file 1: Table S1.** Prediction of repeating sequences in the SJP-SNU genome. **Table S2.** Prediction of non-coding RNAs.

The genome sequence of SJP-SNU.
