# Supplementary material for: Genomic and probiotic characterization of SJP-SNU strain of Pichia kudriavzevii
Source: AMB Express. 2018 May 17;8:80. doi: 10.1186/s13568-018-0609-0 (PMC5957016; doi:10.1186/s13568-018-0609-0)
Supplement: Supplementary file 1 — Additional file 1: Table S1. Prediction of repeating sequences in the SJP-SNU genome. Table S2. Prediction of non-coding RNAs. [file 13568_2018_609_MOESM1_ESM.docx]

**Table S1.** Prediction of repeating sequences in the SJP-SNU genome.

| Type | Length (bp) | (%) in genome |
| --- | --- | --- |
| Retrotransposons | 91,436 bp | 0.83% |
| DNA transposons | 0 bp | 0.00% |
| Unclassified | 17,950 bp | 0.16% |
| Simple repeats | 176,025 bp | 1.60% |
| Total | 315,695 bp | 2.87% |

Table S2. Prediction of non-coding RNAs.

| Type |  | Copy(w) | Ave. length(bp) | Total  length(bp) | % of  genome |
| --- | --- | --- | --- | --- | --- |
| rRNA |  | 29 | - | 34,445 | 0.32 |
|  | 5S rRNA | 6 | 117 | 702 | 0 |
|  | 5.8S rRNA | 6 | 378 | 2,268 | 0.02 |
|  | 18S rRNA | 7 | 1,765 | 12,355 | 0.11 |
|  | 26S rRNA | 6 | 3,070 | 18,420 | 0.17 |
|  | 12S rRNA | 0 | 0 | 0 | 0 |
|  | 16S rRNA | 4 | 175 | 700 | 0.01 |
| tRNA |  | 209 | 76 | 15,899 | 0.14 |
| snRNA |  | 257 | 82 | 21,315 | 0.19 |
|  | CD-box | 202 | 69 | 13,972 | 0.13 |
|  | HACA-box | 39 | 141 | 5,502 | 0.05 |
|  | scaRNA | 4 | 132 | 530 | 0 |
|  | splicing | 10 | 119 | 1194 | 0.01 |
